# Functional and pathological characteristics of reversible remodeling in a canine right ventricle in response to volume overloading and volume unloading

**DOI:** 10.1007/s00595-014-0847-y

**Published:** 2014-02-13

**Authors:** Kazuhiko Ishimaru, Shigeru Miyagawa, Satsuki Fukushima, Haruki Ide, Takaya Hoashi, Toshiharu Shibuya, Takayoshi Ueno, Yoshiki Sawa

**Affiliations:** 1 Department of Cardiovascular Surgery, Osaka University Graduate School of Medicine, 2-2 Yamadaoka, Suita, 565-0871 Japan; 2 Department of Molecular Genetics, Osaka University Graduate School of Medicine, Suita, Japan; 3 Department of Pediatric Cardiovascular Surgery, National Cerebral and Cardiovascular Center, Suita, Japan

**Keywords:** Ventricular remodeling, Valvular disease, Tetralogy of Fallot

## Abstract

**Purposes:**

Patients who undergo right ventricular (RV) outflow augmentation inevitably develop RV remodeling due to pulmonary insufficiency-related volume overload (VOL). However, the reversibility of this remodeling is not fully understood. The goal of this study was to establish an animal model of VOL and unloading to characterize the functional and pathological characteristics and reversibility of RV remodeling.

**Methods:**

VOL-RV was successfully induced by establishing direct RV-pulmonary artery (PA) bypass for 12 weeks in beagle canines. There were no procedure-related mortalities (*n* = 8).

**Results:**

The RV developed typical functional features of VOL-related remodeling, such as a significant increase in end-diastolic/systolic volume and end-systolic pressure and a significant reduction in ejection fraction at 12 weeks, as assessed by three-dimensional echocardiography and cardiac catheterization. The RV developed typical pathological signs of remodeling, microstructural disorganization of cardiomyocytes, and/or structural/functional deterioration of the mitochondria. Volume unloading by division of the RV-PA bypass reversed the increase in the end-systolic/diastolic volume over 4 weeks when compared with a sham operation (*n* = 4 each). In addition, the bypass division also reversed the pathological changes seen in VOL-RV.

**Conclusions:**

VOL-RV that yielded typical functional and pathological features of RV remodeling was reproducibly achieved by direct RV-PA bypass in canines. The RV remodeling due to VOL was functionally and pathologically reversed by volume unloading via the bypass division.

## Introduction

Surgical augmentation of congenitally stenotic outflow of the right ventricle (RV) yields favorable clinical outcomes. However, most patients undergoing this procedure ultimately develop volume overload (VOL) of the RV due to pulmonary valve insufficiency [1]. Prolonged VOL results in progressive functional deterioration of the RV, eventually leading to end-stage RV failure in the absence of surgical intervention [2]. Mechanisms underlying the development of RV failure and the optimal timing for surgical intervention have not been definitively determined [3].

Left ventricle (LV) VOL secondary to aortic valve insufficiency induces LV remodeling, in which cardiac fibroblasts are activated to modulate the extracellular matrix [4] and in which cardiomyocytes develop mitochondrial dysfunction-associated pathological hypertrophy [5, 6]. This leads to dilation of the LV cavity and impairment of systolic and diastolic LV function. Volume-unloading surgery, such as aortic valve replacement, can pathologically and functionally reverse VOL-induced LV remodeling [4]. Mitochondrial dysfunction in cardiomyocytes is an early predictor of irreversible LV remodeling and may therefore be an indication for surgical intervention for VOL [7].

Pulmonary valve replacement may also reverse the RV remodeling associated with pulmonary valve insufficiency [1]. However, the pathologic mechanism by which this occurs is poorly understood. Investigators have previously developed animal models of VOL-RV [8], but volume unloading is not feasible in those models, therefore precluding pathologic study of reverse RV remodeling. Thus, the goal of the present study was to develop a novel reproducible canine model of VOL-RV and to test the hypothesis that VOL-induced RV remodeling is functionally and pathologically reversible through a volume-unloading surgical intervention.

## Materials and methods

Animal studies were performed with the approval of the institutional ethics committee. All protocols conformed to the Principles of Laboratory Animal Care formulated by the National Society for Medical Research and the Guide for the Care and Use of Laboratory Animals (US National Institutes of Health Publication No. 85-23, revised 1996).

### RV volume overloading and unloading model

**Fig. 1:**
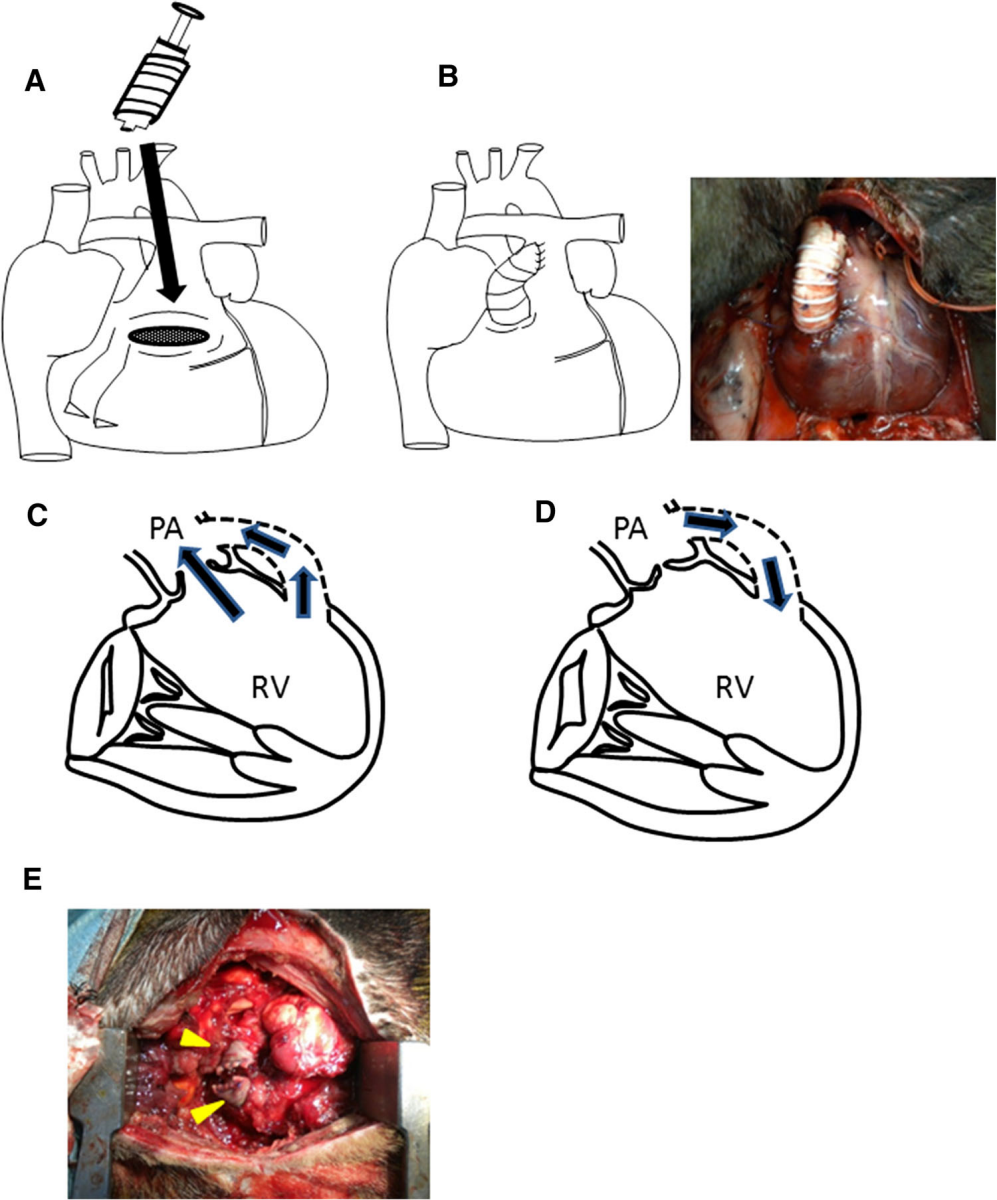
Volume-overloaded right ventricle (VOL-RV) model was generated by placing a conduit between the RV and pulmonary artery (PA) using a vascular graft (**a**, **b**). RV-PA bypass produced antegrade/retrograde blood flow in the systolic/diastolic phase (**c**, **d**), mimicking the hemodynamics of pulmonary valve insufficiency. The conduit was divided (*arrowhead*) 12 weeks later to unload VOL-RV, mimicking pulmonary valve replacement (**e**)

A canine model of RV-pulmonary artery (PA) conduit bypass model was established to create RV volume overload without mechanical circulatory support (Fig. 1a, b). Under monitoring of the electrocardiogram, eleven-week-old male Toyo beagle canines (Oriental Yeast, Japan, *n* = 8) were anesthetized with intravenous administration of propofol (2–4 mg/kg/h) after a intramuscular injection of xylazine (5 mg/kg) and followed by intubation and inhalation anesthesia with 1.5–2.5 % sevoflurane delivered through a volume-controlled respirator (200 ml, 12 cycles/min) with room air.

A left thoracotomy was performed at the fourth intercostal space, and the main PA was carefully exposed. We modified the RV-PA technique using a 10-mm ring-enforced vascular graft consistent with the pulmonary trunk (PT) diameter [9]. A purse-string suture was made with 5-0 polypropylene suture transversely at the RV outflow tract, and transverse ventriculotomy was performed. Controlling the bleeding from RV ventriculotomy, a 10-mm ring-enforced graft (Gore-Tex, Japan), which was inserted in the 2.5-ml syringe for preservation of the graft formation, was carefully inserted toward the RV apex (Fig. 1a). Following the removal of the syringe, care was taken to ensure that approximately a half of the PT above the annulus was attained within the partial clamp. A longitudinal incision was made on the PT; the graft was anastomosed after trimming the length of the graft (Fig. 1b–d). After removal of the clamp, the thoracotomy was closed in a layered fashion.

The beagles were then housed in temperature-controlled individual cages for 12 weeks. At that time, they underwent re-do thoracotomy, canines with VOL-RV were randomly assigned to one of two groups: RV-PA bypass division mimicking pulmonary valve replacement (overloading–unloading group: O–U group, *n* = 4) or sham operation (overloading group: O group, *n* = 4, Fig. 1e).

**Fig. 2:**
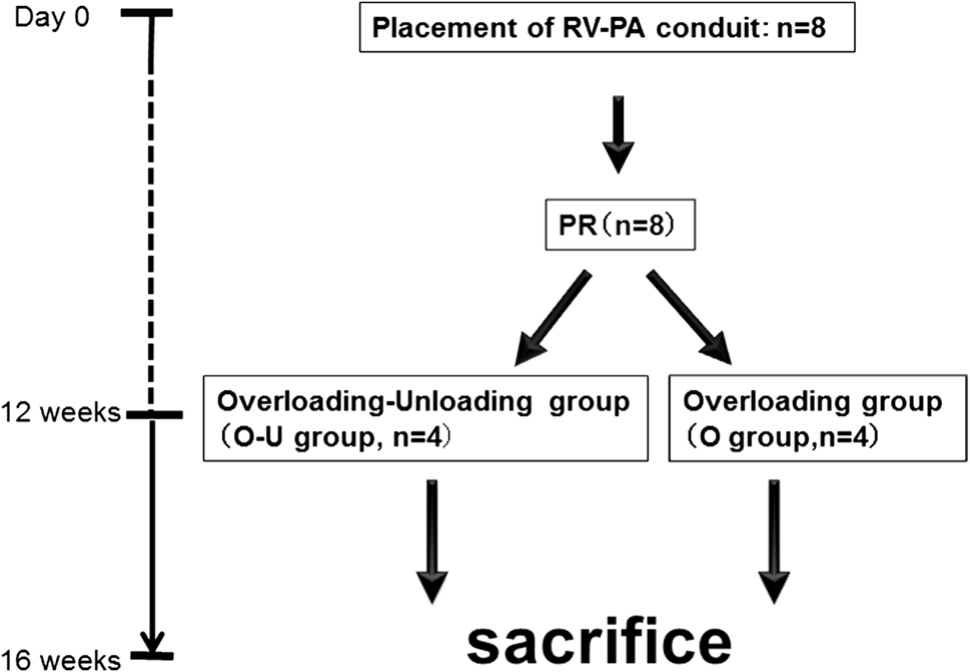
Study protocol. *PA* pulmonary artery, *PR* pulmonary regurgitation, *RV* right ventricle

Following recovery for 4 weeks, animals were humanely killed by deep inhalational anesthesia (5 % sevoflurane) followed by intravenous infusion of potassium chloride (10 mmoL), and their hearts were promptly excised (Fig. 2).

### Transthoracic Doppler and three-dimensional echocardiography

Transthoracic echocardiography was performed under inhalational anesthesia (1.5 % sevoflurane) to acquire short-axis and four-chamber views with the Intelligent Echocardiography System 33 (Philip Electronics Japan, Ltd.). Real-time three-dimensional echocardiography was then performed using an X3-1 Matrix-Array probe (Philips Electronics Japan, Ltd) to visualize the RV from the four-chamber view. The three-dimensional RV images were then reconstructed (Echo View, Tomtec Imaging Systems, Munich, Germany) to calculate end-diastolic/systolic volumes (EDV/ESV) and ejection fraction (EF) [10]. These cardiac parameters were assessed every 4 weeks after RV-PA bypass to evaluate functional relevancy of this model to VOL-RV in the clinical settings.

### Cardiac catheterization

A conductance catheter (Unique Medical, Tokyo, Japan) was placed in the RV apex through the main PA with animals under general anesthesia. A Millar 5 Fr pressure-tip catheter (Unique Medical) was also placed in the RV cavity through the anterior wall. Pressure–volume loops and intracardiac electrocardiograms were monitored to measure end-systolic/diastolic pressure (ESP/EDP), heart rate (HR), and d*p*/d*t* max/min (Integral 3, Unique Medical). Subsequently, end-systolic/end-diastolic pressure–volume relationships (ESPVR/EDPVR) were measured by occluding the inferior vena cava [11]. Functional impacts of the VOL and volume-unloading intervention were assessed by cardiac catheterization at 12 weeks after RV-PA bypass and at 4 weeks after the bypass division or sham operation.

### Biomarker and tissue weight

Venous blood was collected every 4 weeks, and the plasma was promptly stored at −80 °C. N-terminal pro-brain natriuretic peptide (NT-pro BNP) level in the plasma was assessed by Cardiopet^®^ (IDEXX Laboratories, Japan). The RV free-wall and interventricular septum was promptly separated from the excised hearts and was blotted dry and weighed.

### Histology

The excised RV free-wall tissue was fixed with 10 % formalin and embedded in paraffin, and cut into 5-μm-thick sections. Sections were stained with H&E to measure short-axis length of the RV cardiomyocytes in the ≥100 fields per slide, including endocardial, epicardial and mid layers, by ImageJ software. Sections were also stained with picro-sirius red. The percentage of red-stained area, corresponding to fibrotic area, was assessed using a computer-based method [12]. The red-stained collagen area was then divided by the total number of pixels in the field. Ten fields of endocardial layers of the RV wall per slide were analyzed and averaged. In addition, changes in pathological features of the VOL-RV in response to the unloading intervention were assessed by measuring the dry weight of the RV after being killed.

### Electron microscopy

Tissue samples were fixed in 30 M 4-(2-hydroxyethyl)-1-piperazineethanesulfonic acid (HEPES) buffer containing 2 % glutaraldehyde at 4 °C and were post-fixed in 2 % OsO_4_ at 4 °C for 3 h and dehydrated by dipping into a graded series of ethanol for 10 min each. Samples were then incubated in propylene oxide for 30 min, followed by incubation in a mixture of propylene oxide and epoxy resin for 1 h. They were subsequently embedded in a gelatin capsule with epoxy resin at 60 °C for 2 days. Ultrathin sections were stained with uranyl acetate and lead citrate (Hanaichi Ultrastructure Research Institute, Aichi, Japan).

### Histochemical analyses of succinate dehydrogenase (SDH) activity

Seven-micrometer-thick cryosections from the RV tissues were incubated with 0.1 mol/l Tris–HCl (pH 7.4), 10 mg/ml sodium succinate, 1 mg/ml Nitroblue tetrazolium and 25 μg/ml phenazine methosulfate at 37 °C for 30 min. Slides were rinsed in water and dehydrated with acetone for assessment under microscopy (AxioImager M1, Zeiss, Germany).

### Real-time polymerase chain reaction (PCR)

RNA was extracted from the RV using the RNeasy Kit (Qiagen) and then was reverse-transcribed using Omniscript reverse transcriptase (Qiagen). Real-time PCR was performed for succinate dehydrogenase (SDH), cytochrome (Cyt)-C, mitochondrially encoded Nicotinamide adenine dinucleotide (NADH) dehydrogenase (mtND)1 and mitochondrially encoded Adenosine Triphosphate (ATP) synthase 6 (mtATP6) using the 7500 Fast real-time PCR system (Applied Biosystems). The quantities of the respective messenger RNAs (mRNAs) were normalized using glyceraldehyde-3-phosphate dehydrogenase (GAPDH) as a control (Table 1).

**Table 1:** Forward and reverse primers in the mitochondrial genes and GAPDH

Assay	Forward	Reverse
*GAPDH*	5′ GTGATGCTGCTGCTGAGTATGTTG 3′	5′ TTGCTAGAGGAGCCAAGCAGTT 3′
*SDH*	5′ CAGCTCTATGGAGACCTAAAGCATCT 3′	5′ TCTGCAGCTCCAGGGTCTCT 3′
*Cyt*-*C*	5′ GGCCCCTGGATTTTCTTACAC 3′	5′ CCAGGGATGTACTTCTTGGGAAC 3′
*mtND1*	5′ AACCCTAGCCATGATATGATTCA 3′	5′ ACTAGTTCGGATTCTCCTTCAGTTAAGT 3′
*mtATP6*	5′ GATCGTCATATTCCCTTCCATTTTA 3′	5′ TGCTGAATGGAGATTAACCGATT 3′

### Statistical analysis

Data are expressed as the mean ± SEM. NT-pro BNP and echocardiographic data were analyzed by two-way repeated analysis of variance (ANOVA) for differences across the entire time course, and one-way ANOVA with the Turkey–Kramer post hoc test was used to verify the significance for specific comparison at each time point. Other data were assessed by the unpaired *t* test. Statistical analysis was performed with SPSS Version 11.0 (SPSS, Chicago, IL, USA). *p* < 0.05 was considered to represent statistical significance.

## Results

### Cardiac parameters in the VOL-RV canine heart

There were no procedure-related mortalities or morbidities for this operation. Doppler echocardiography detected blood flow through the bypass conduit in both the systolic and diastolic phases with competent pulmonary valve in all canines after the RV-PA bypass was established.

**Fig. 3:**
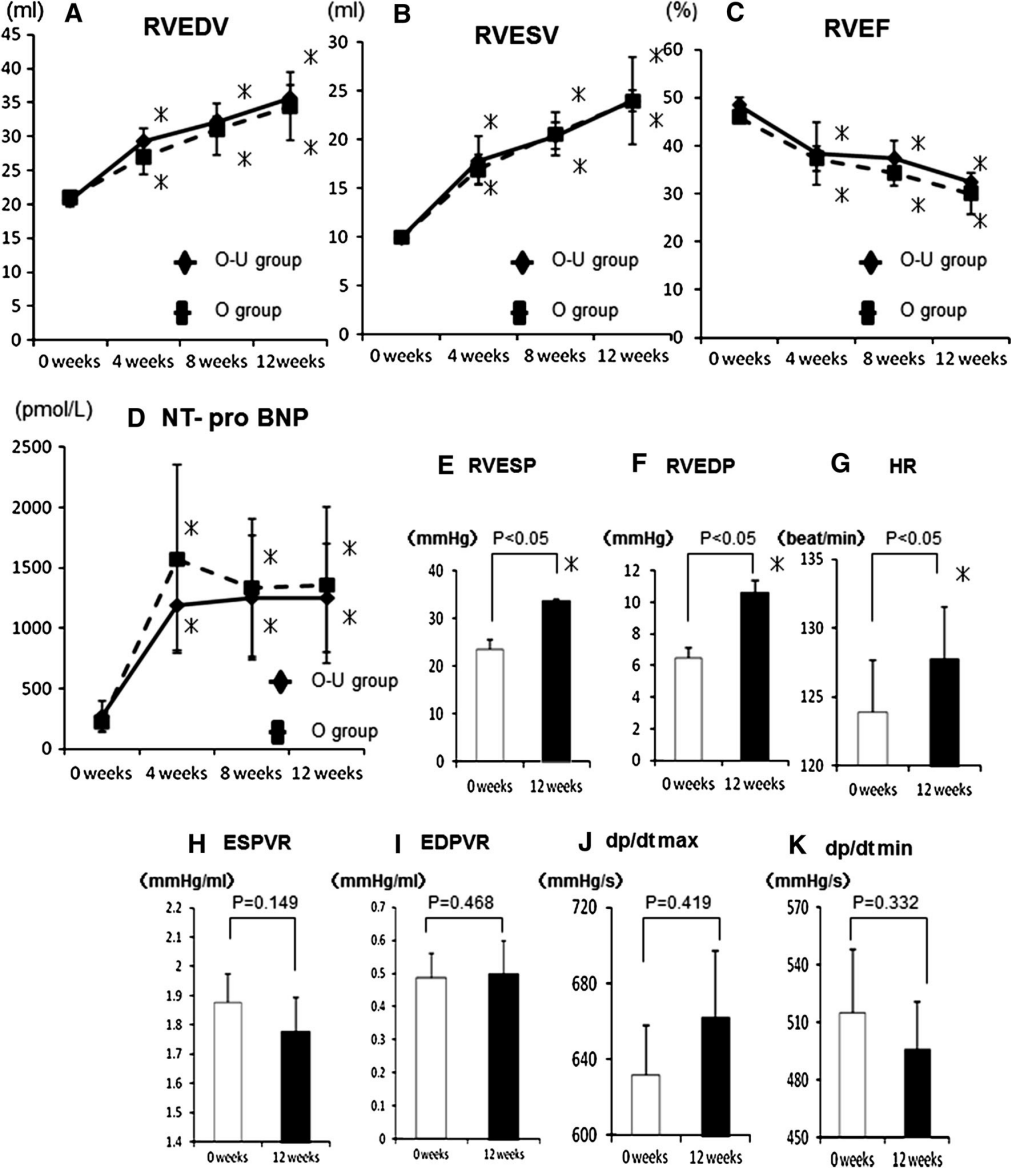
Right ventricular end-diastolic/systolic volume (RVEDV/ESV, **a**, **b**) and RV ejection fraction (RVEF, **c**) assessed by three-dimensional echocardiography, and plasma N-terminal pro-brain natriuretic peptide (NT-pro BNP) levels after the bypass were serially measured (**d**). Hemodynamic indices; right ventricular end-systolic/diastolic pressure (RVESP/EDP, **e**, **f**), heart rate (HR, **g**), end-systolic/end-diastolic pressure–volume relationships (ESPVR/EDPVR, **h**, **i**), *d*p/*d*t max/min (**j**, **k**) in the normal RV or VOL-RV at 12 weeks after the bypass. *p < 0.05 versus 0 weeks

Three-dimensional echocardiography showed a progressive increase in EDV and ESV and a progressive reduction of EF over 12 weeks (Fig. 3a–c). Plasma NT-pro BNP levels significantly increased after the RV-PA bypass (Fig. 3d). ESP, EDP and HR, assessed by cardiac catheterization, were significantly elevated in animals with VOL-RV when compared with animals with normal hearts (Fig. 3e–g), whereas there were no significant differences in other parameters when comparing the two groups (Fig. 3h–k). Regurgitation of the tricuspid valve was absent throughout the study period.

### Histopathological changes in the VOL-RV canine heart

**Fig. 4:**
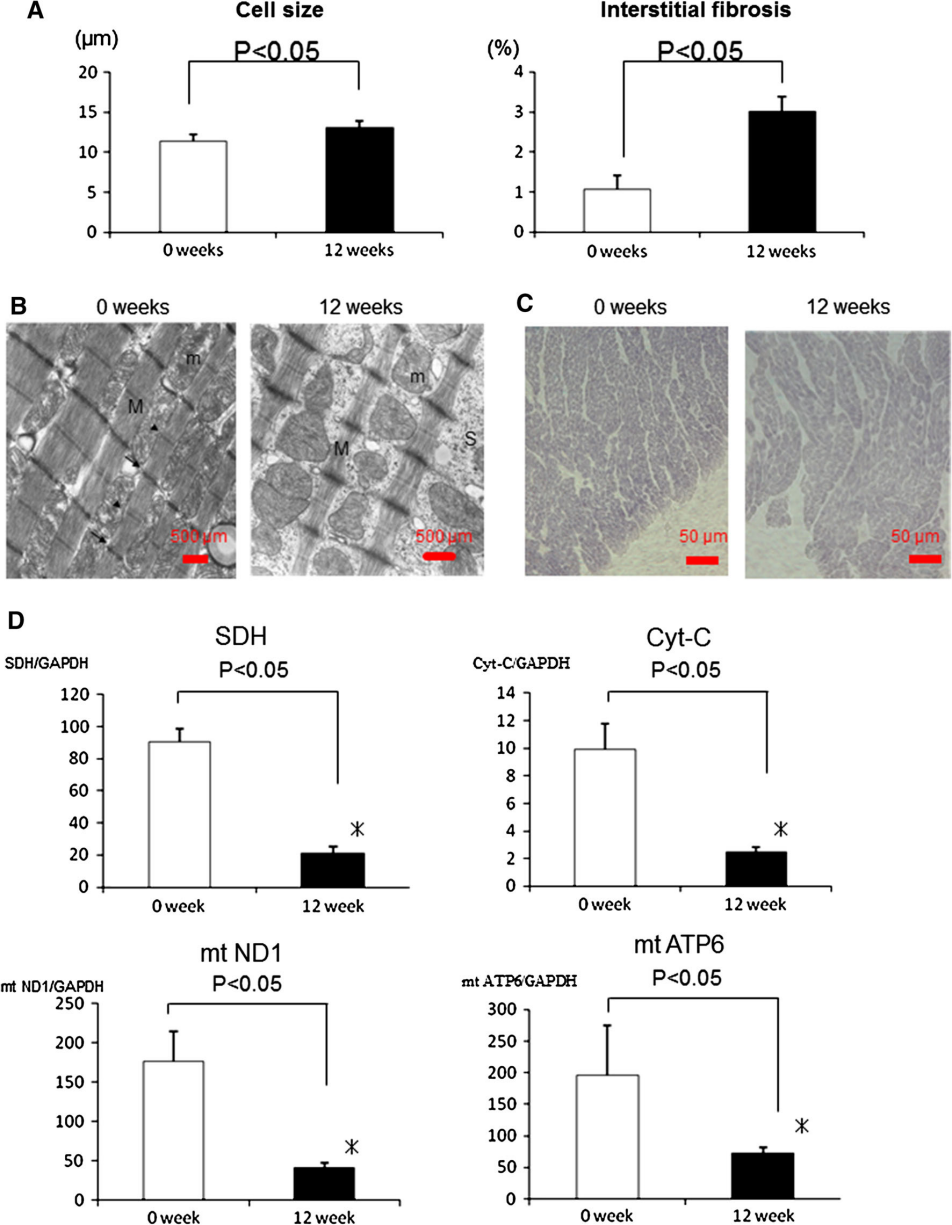
Size of the RV cardiomyocytes and quantity of interstitial collagen in the volume-overloaded right ventricle (VOL-RV) and normal RV (**a**). Electron microscopy showed widened sarcoplasmic spaces between the myofibrils. In addition, the myofilament (M) Z line (*arrow*) and M lines (*arrow head*) were disorganized, and the mitochondria (m) showed polymorphic structure in the VOL-RV when compared with the normal RV (**b**). Succinate dehydrogenase (SDH) activity was homogeneously downregulated in the VOL-RV when compared with that in the normal heart (**c**). mRNA level of the mitochondrial cellular respiration-related genes in the VOL-RV and the normal heart (**d**); cytochrome *C* (Cyt-C), mitochondrially encoded NADH dehydrogenase 1 (mtND1), mitochondrially encoded ATP synthase 6 (mtATP6) and glyceraldehyde-3-phosphate dehydrogenase (GAPDH). *p < 0.05 versus 0 weeks

Size of the RV cardiomyocytes, assessed by H&E, was significantly greater in VOL-RV hearts when compared with normal hearts (*p* < 0.05, Fig. 4a). Interstitial fibrosis, assessed by picro-sirius red staining, was homogeneously present in the RV free-wall or VOL-RV hearts only minimally so in the normal RV. Quantity of interstitial fibrosis, assessed by computer-based morphometry, was significantly greater in the VOL-RV hearts when compared with normal hearts (*p* < 0.05, Fig. 4a).

### Microstructure and mitochondrial function in the VOL-RV canine heart

Electron microscopy showed that sarcoplasmic spaces were widened between the myofibrils in the VOL-RV, whereas those in the normal heart were tightly filled with myofibrils and mitochondria (Fig. 4b); in addition, the myofilaments were disorganized and the mitochondria showed polymorphic structure in the VOL-RV when compared with that in the normal heart (Fig. 4b). SDH enzyme activity, representing mitochondrial function, was globally and homogeneously downregulated in the VOL-RV when compared with that in the normal heart (Fig. 4c). In addition, mRNA levels of mitochondrial cellular respiration-related genes, such as SDH, cyt-C, mtND1 and mtATP6, were significantly and markedly decreased in the VOL-RV when compared with that in the normal heart (Fig. 4d).

### Functional recovery after volume-unloading intervention

**Fig. 5:**
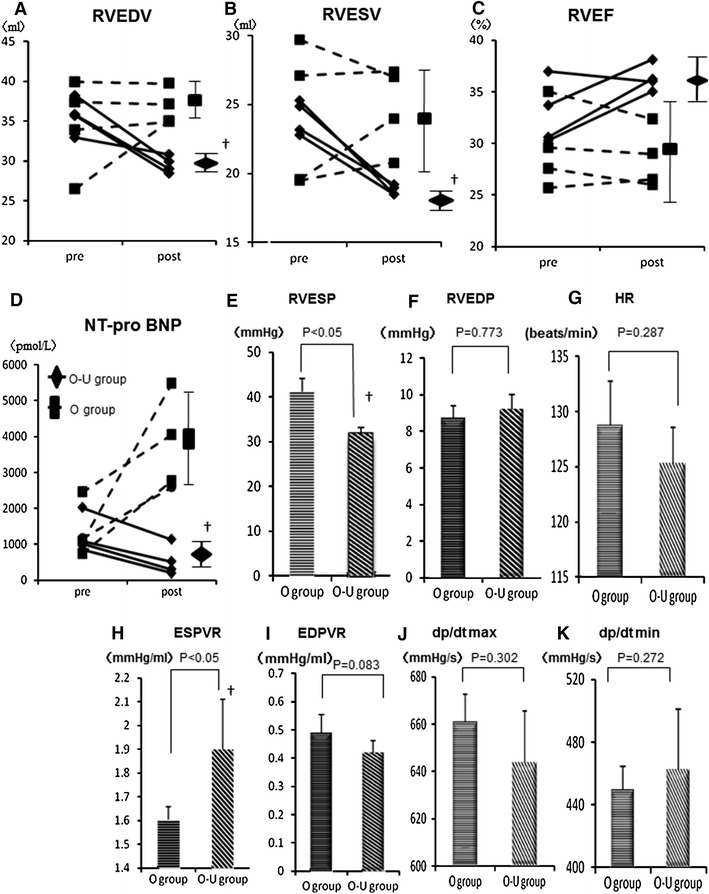
Three-dimensional echocardiographic parameters. Right ventricular end-diastolic/systolic volume (RVEDV/ESV, **a**, **b**), right ventricular ejection fraction (RVEF, **c**) and plasma N-terminal pro-brain natriuretic peptide (NT-pro BNP, **d**) levels before and after re-intervention. Hemodynamic indices; right ventricular end-systolic/diastolic pressure (RVESP/EDP, **e**, **f**), heart rate (HR, **g**), end-systolic/end-diastolic pressure–volume relationships (ESPVR/EDPVR, **h**, **i**), d*p*/d*t* max/min (**j**, **k**) at 4 weeks after re-intervention. ^†^ *p* < 0.05 versus O group

EDV and ESV were significantly smaller in the overloading–unloading (O–U) group than in the overloading (O) group or relative to baseline values taken prior to bypass division (*p* < 0.05, Fig. 5a–c). Plasma NT-pro BNP levels and ESP were significantly lower in the O-U group than in the O group (*p* < 0.05, Fig. 5d, e), but there were no differences in EDP or HR when comparing the two groups (Fig. 5f, g). ESPVR was significantly greater in the O-U group than in the O group (*p* < 0.05, Fig. 5h), but there was no significant difference in EDPVR, d*p*/d*t* max, and d*p*/d*t* min when comparing the two groups (Fig. 5i–k).

### Changes in pathological features in response to volume-unloading intervention

**Fig. 6:**
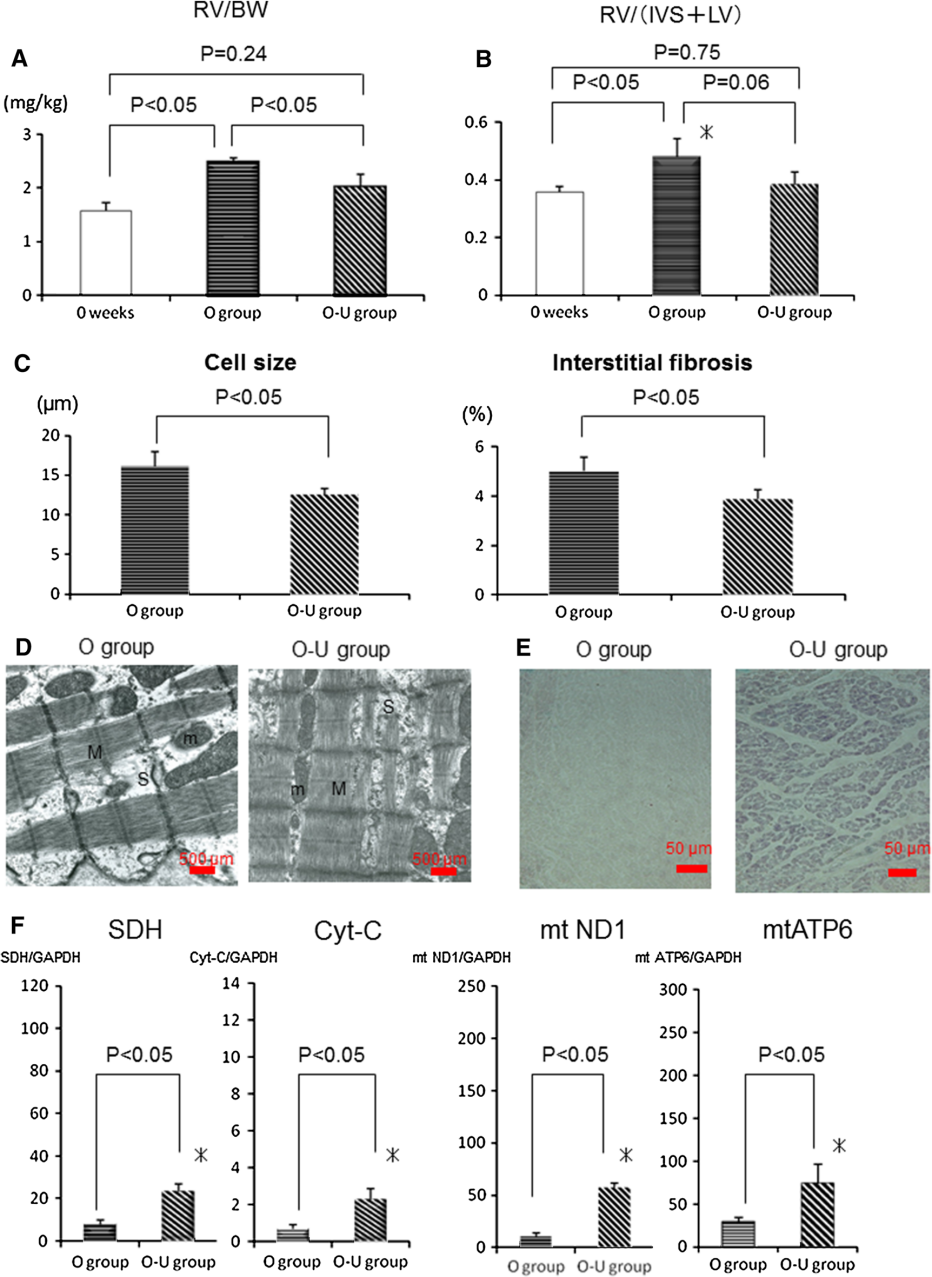
Body weight (BW) of the right ventricle (RV, **a**) and weight ratios of the RV, interventricular septum (IVS) and left ventricle **(**LV**, b**) were assessed in normal hearts and in the overloading (O) and overloading–unloading (O–U) groups. Cardiomyocyte size and interstitial collagen (**c**) were assessed in both groups. Electron microscopy showed widened sarcoplasmic spaces between the myofibrils (M) and polymorphic mitochondria (m) in the O group and tightly filled sarcoplasmic spaces and normalized mitochondrial structure in the O-U group (**d**). Succinate dehydrogenase (SDH) enzyme activity (**e**) and real-time polymerase chain reaction (**f**) were performed for mitochondrial cellular respiration-related genes in both groups; cytochrome *C* (Cyt-C), mitochondrially encoded NADH dehydrogenase 1 (mtND1), mitochondrially encoded ATP synthase 6 (mtATP6) and glyceraldehyde-3-phosphate dehydrogenase (GAPDH). *p < 0.05 versus O group

RV/BW dry-weight ratio in the O–U group was not significantly greater than that in the normal heart (*p* = 0.24) but was significantly lower than that in the O group (*p* < 0.05, Fig. 6a). RV/(LV + IVS) dry-weight ratio in the O–U group was not significantly different from that in the normal heart (*p* = 0.75, Fig. 6b) but was lower than those in the O group (*p* = 0.06, Fig. 6b). Size of the RV cardiomyocytes was significantly smaller in the O–U group than in the O group (*p* < 0.05). Quantity of collagen in the RV interstitium was significantly less in the O–U group than in the O group (Fig. 6c).

### Changes in microstructure and mitochondrial function in response to volume-unloading intervention

Electron microscopy showed that sarcoplasmic spaces between the myofibrils in the cardiomyocytes were widened in the O group and were tightly filled with the myofibrils and mitochondria in the O–U group (Fig. 6d). The reduction of SDH enzyme activity was accelerated in the O group, but not at 4 weeks in the O–U group (Fig. 6e). Further, mRNA levels of the mitochondria-related molecules were significantly higher in the O–U group than in the O group (Fig. 6f).

## Discussion

### Summary of findings

The present study established a reproducible canine model of VOL-RV by performing RV-PA shunt. These animals developed typical features of pulmonary valve insufficiency-induced RV failure, such as progressive dilatation of the RV cavity, progressive deterioration of RV function, pathological hypertrophy, accumulation of interstitial fibrosis, disorganized myofilaments, mitochondrial dysfunction, and elevation of plasma NT-pro BNP levels. Functional and pathological features of the VOL-RV in this model, including microstructure of the cardiomyocytes and mitochondrial function, were at least partially reversed by a volume-unloading intervention (i.e., division of the RV-PA bypass).

### RV volume overloading/unloading model

A large number of patients who undergo repair of RV outflow obstruction develop progressive RV failure due to pulmonary valve insufficiency-induced VOL [13]. However, optimal timing of the surgical repair for the VOL-RV has not been determined, at least in part due to the lack of reproducible model of VOL-RV and unloading. While several models of VOL-RV have been previously described [8, 14, 15], the present study used direct RV-PA bypass and subsequent division of RV-PA bypass to establish a VOL-RV and unloading model with minimal procedure-related mortalities. Of note, the RV in this model developed physiological and pathological changes in a consistent manner, indicating a reproducible and appropriate model of VOL-RV and unloading.

### Functional and pathological relevancy of the VOL-RV canine model

In this study, we used three-dimensional echocardiography to assess RV volume and function, which correlated well with that by cardiac magnetic resonance in clinical practice [9]. In the present model, there was a progressive dilation of RV volume and a reduction in the RV ejection fraction, which is typical of the functional changes seen in the patients with VOL-RV after tetralogy of Fallot correction [2, 3]. In addition, cardiac catheterization showed progressive elevation of both end-systolic, end-diastolic pressures and HR but no significant change in ESPVR, EDPVR, d*p*/d*t* max, d*p*/d*t* min or Tau over the 12-week study period. These findings are consistent with those reported by Agger et al. [14] who induced VOL-RV in pigs via external plication of the pulmonary valve/annulus for 10 weeks. By contrast, Kuehne et al. [15] induced VOL-RV in growing swine model by transcatheter placement of stents across the pulmonary valve for 12 weeks; this resulted in a decrease in ESPVR, but no change in EDPVR. These different findings might be explained by various factors, including differences in animal size, techniques, and study durations. In the present model, abnormalities in mitochondrial structure and cellular respiration-related gene expression were present in RV tissue from all VOL animals. In addition, d*p*/d*t* max or min, which represents systolic/diastolic function, was impaired in this model, possibly because these parameters are modulated by both contractile function and preloading conditions [16]. RVESP significantly increased in this VOL-RV model, despite the absence of infundibular, valvular or supravalvular stenosis, which might be explained by eccentric and concentric hypertrophy induced by VOL, similar to hypertrophy seen in the context of aortic valve regurgitation [17].

Plasma NT-pro BNP level is a sensitive indicator of RV function after TOF correction [18]. In the present model, NT-pro BNP level increased after RV-PA bypass, suggesting a clinically relevant response to VOL-RV. In the O group, NT-pro BNP level was elevated at 4 weeks after sham operation; this elevation may be due to LV dysfunction mediated by the RV–LV interaction [19].

The relationship between cardiac performance and histological/biochemical parameters in the VOL-RV has not been investigated, but reversibility of aortic insufficiency-induced LV failure correlates with recovery in the activity of mitochondrial enzymes, such as ATPase or SDH [4]. Interestingly, Modesti et al. [20] reported that, due to differences in the embryonic origin between the RV and the LV, the RV appeared more prone to develop interstitial fibrosis in the setting of VOL. This suggests that mitochondrial dysfunction might precede irreversible RV remodeling and might be a sensitive indicator of the reversibility of VOL-RV. Diastolic RV function was preserved despite VOL in this study, which might be due to the highly adaptable nature of the RV against physiological changes, including VOL [20, 21].

### Effects of volume-unloading on VOL-RV in the canine model

A volume-unloading intervention, such as pulmonary valve replacement, can functionally reverse RV remodeling in clinical practice, but the mechanisms underlying this process remain poorly understood. In this study, a volume-unloading intervention elicited recovery of systolic RV function over 4 weeks. Such immediate recovery of RV contractility after the unloading intervention may result from improvement in energy supply and improvement in the demand ratio associated with recovery of mitochondria. This functional recovery was associated with reverse pathological remodeling, including reversal of cardiomyocyte hypertrophy, interstitial fibrosis, and/or mitochondrial dysfunction. In addition, plasma NT-pro BNP levels decreased following volume unloading. These findings suggest that an optimally timed volume-unloading intervention might fully reverse VOL-induced RV remodeling.

### Limitations

This study was limited by the use of an animal model and the small number of cases [4] in each group, in which RV-PA bypass was established to induce VOL mimicking pulmonary valve insufficiency. In addition, volume unloading was achieved by the bypass division, though an insufficient pulmonary valve is repaired by valve replacement in the clinical setting. Therefore, the behavior of the RV in response to VOL and unloading in this study may not be fully consistent and quantitative in comparison to that in the clinical setting. However, the functional and pathological trends, which were proven to be consistent, are considered to be relevant to the clinical setting. Finally, no procedure-related mortalities were observed.

## Conclusions

VOL-RV induced by direct RV-PA bypass in canines reproducibly yielded typical functional and pathological features of RV remodeling, including hypertrophy of the RV cardiomyocytes, interstitial fibrosis and structural and functional deterioration of the mitochondria. The RV remodeling due to VOL was functionally and pathologically reversed by volume unloading via division of the bypass.

## References

[1] Gatzoulis MA, Balaji S, Webber SA, Siu SC, Hokanson JS, Poile C (2000). Risk factors for arrhythmia and sudden cardiac death late after repair of tetralogy of Fallot: a multicentre study. Lancet.

[2] Harrison DA, Harris L, Siu SC, MacLoghlin CJ, Connelly MS, Webb GD (1997). Sustained ventricular tachycardia in adult patients late after repair of tetralogy of Fallot. J Am Coll Cardiol.

[3] Therrien J, Siu SC, McLaughlin PR, Liu PP, Williams WG, Webb GD (2000). Pulmonary valve replacement in adults late after repair tetoralogy of Fallot: are we operating too late?. J Am Coll Cardiol.

[4] Donaldson RM, Florio R, Richards AF, Bennett JG, Yacoub M, Ross DN (1982). Irreversible morphological changes contributing to depressed cardiac function after surgery for chronic aortic regurgitation. Br Heart J.

[5] Wang X, Ren B, Liu S, Sentex E, Tappia PS, Dhalla NS (2002). Characterization of cardiac hypertrophy and heart failure due to volume overload in the rat. J Appl Physiol.

[6] Ozcan C, Bienengraeber M, Hodgson DM, Mann DL, Terzic A (2003). Mitochondrial tolerance to stress impaired in failing heart. J Mol Cell Cardiol.

[7] Oliver-Dussault C, Ascah A, Marcil M, Matas J, Picard S, Pibarot P (2010). Early predictors of cardiac decompensation in experimental volume overload. Mol Cell Biochem.

[8] Yerebankan C, Klopsch C, Prietz S, Boltze J, Vollmar B, Liebold A (2009). Pressure-volume loop: feasible for the evaluation of right ventricular function in an experimental model of acute pulmonary regurgitation?. Interact Cardiovasc Thorac Surg.

[9] Schreiber C, Kasnar-Samprec J, Eicken A, Cleuziou J, Prodan Z, Lange R (2009). Ring-enforced right ventricle-to-pulmonary artery conduit in Norwood stage I reduces proximal conduit stenosis. Ann Thorac Surg.

[10] Gopal AS, Chukwu EO, Katz AS, Toole RS, Schapiro W, Reichek N (2007). Normal values of right ventricular size and function by real-time 3-dimensional echocardiography: comparison with cardiac resonance imaging. J Am Soc Echocardiogr.

[11] Sato T, Shishido T, Inagaki M, Miyashita H, Sugimachi M, Sunagawa K (1998). ESPVR of in situ rat left ventricle shows contractility-dependent curvilinearity. Am J Physiol Heart Circ Physiol.

[12] Hoashi T, Matsumiya G, Miyagawa S, Ueno T, Okano T, Sawa Y (2009). Skeletal myoblast sheet transplantation improves the diastolic function of a pressure-overloaded right heart. J Thorac Cardiovasc Surg.

[13] Adamson L, Vohra HA, Haw MP (2009). Does pulmonary valve replacement post repair tetralogy of Fallot improve right ventricular function?. Interact Cardiovasc Thorac Surg.

[14] Agger P, Hyldebrandt JA, Nielsen EA, Hjortdal V, Smerup M (2010). A novel porcine model for right ventricular dilatation by external suture plication of the pulmonary valve leaflets. Interact Cardiovasc Thorac Surg.

[15] Kuehne T, Saeed M, Turner D, Higgins CB, Moore P (2003). Effects of pulmonary insufficiency on biventricular function in the developing heart of growing swine. Circulation.

[16] Little WC, Cheng CP, Mumma M, Igarashi Y, Johansen JV, Johnston WE (1989). Comparison of measures of left ventricular contractile performance derived from pressure-volume loops in conscious dogs. Circulation.

[17] Wisenbaugh T, Spann JF, Carabello BA (1984). Differences in myocardial performance and load between patients with similar amounts of chronic aortic versus chronic mitral regurgitation. J Am Coll Cardiol.

[18] Randostaw P, Bozena W (2009). Usefulness of NT-proBNP in assessment of right ventricular function in children after tetralogy of Fallot correction-a preliminary study. Kardiol Pol..

[19] Kempny A, Diller GP, Orwat S, Kaleschke G, Ach Bunck, Maintz D (2012). Right ventricular–left ventricular interaction in adults with tetralogy of Fallot: a combined cardiac magnetic resonance and echocardiographic speckle tracking study. Int J Cardiol.

[20] Modesti PA, Vanni S, Bertolozzi I, Cecioni I, Lumachi C, Perma AM (2004). Different growth factor activation in the right and left ventricles in experimental volume overload. Hypertension.

[21] De Stefano LM, Matsubara LS, Matsubara BB (2006). Myocardial dysfunction with increased ventricular compliance in volume overload hypertrophy. Eur J Heart Fail.

